# Inhibition of TGF-β Signaling Enables Human Corneal Endothelial Cell Expansion *In Vitro* for Use in Regenerative Medicine

**DOI:** 10.1371/journal.pone.0058000

**Published:** 2013-02-25

**Authors:** Naoki Okumura, EunDuck P. Kay, Makiko Nakahara, Junji Hamuro, Shigeru Kinoshita, Noriko Koizumi

**Affiliations:** 1 Department of Biomedical Engineering, Faculty of Life and Medical Sciences, Doshisha University, Kyotanabe, Japan; 2 Department of Ophthalmology, Kyoto Prefectural University of Medicine, Kyoto, Japan; University of Reading, United Kingdom

## Abstract

Corneal endothelial dysfunctions occurring in patients with Fuchs' endothelial corneal dystrophy, pseudoexfoliation syndrome, corneal endotheliitis, and surgically induced corneal endothelial damage cause blindness due to the loss of endothelial function that maintains corneal transparency. Transplantation of cultivated corneal endothelial cells (CECs) has been researched to repair endothelial dysfunction in animal models, though the *in vitro* expansion of human CECs (HCECs) is a pivotal practical issue. In this study we established an optimum condition for the cultivation of HCECs. When exposed to culture conditions, both primate and human CECs showed two distinct phenotypes: contact-inhibited polygonal monolayer and fibroblastic phenotypes. The use of SB431542, a selective inhibitor of the transforming growth factor-beta (TGF-β) receptor, counteracted the fibroblastic phenotypes to the normal contact-inhibited monolayer, and these polygonal cells maintained endothelial physiological functions. Expression of ZO-1 and Na^+^/K^+^-ATPase maintained their subcellular localization at the plasma membrane. Furthermore, expression of type I collagen and fibronectin was greatly reduced. This present study may prove to be the substantial protocol to provide the efficient *in vitro* expansion of HCECs with an inhibitor to the TGF-β receptor, and may ultimately provide clinicians with a new therapeutic modality in regenerative medicine for the treatment of corneal endothelial dysfunctions.

## Introduction

Corneal endothelial dysfunction is a major cause of severe visual impairment leading to blindness due to the loss of endothelial function that maintains corneal transparency. Restoration to clear vision requires either full-thickness corneal transplantation or endothelial keratoplasty. Recently, highly effective surgical techniques to replace corneal endothelium [e.g., Descemet's stripping automated endothelial keratoplasty (DSAEK) and Descemet's membrane endothelial keratoplasty (DMEK)] have been developed [1]–[3] that are aimed at replacing penetrating keratoplasty for overcoming pathological dysfunctions of corneal endothelial tissue. At present, our group and several other research groups have focused on the establishment of new treatment methods suitable for a practical clinical intervention to repair corneal endothelial dysfunctions [4]–[9]. Since corneal endothelium is composed of a monolayer and is a structurally flexible cell sheet, corneal endothelial cells (CECs) have been cultured on substrates including collagen sheets, amniotic membrane, or human corneal stroma. Then the cultured CECs are transplanted as a cell sheet. However, these techniques require the use of an artificial or biological substrate that may introduce several problems such as substrate transparency, detachment of the cell sheet from the cornea, and technical difficulty of transplantation into the anterior chamber. In our effort to overcome those substrate-related problems, we previously demonstrated that the transplantation of cultivated CECs in combination with a Rho kinase (ROCK) inhibitor enhanced the adhesion of injected cells onto the recipient corneal tissue without the use of a substrate and successfully achieved the recovery of corneal transparency in two corneal-endothelial-dysfunction animal models (rabbit and primate) [10], [11].

However, in the context of the clinical setting, another pivotal practical issue is the *in vitro* expansion of human CECs (HCECs). HCECs are vulnerable to morphological fibroblastic change under normal culture conditions. Although HCECs can be cultivated into a normal phenotype maintaining the contact-inhibited polygonal monolayer, they eventually undergo massive endothelial-mesenchymal transformation after long-term culture or subculture. Thus, cultivation of HCECs with normal physiological function is difficult, yet not impossible [12], [13].

Epithelial mesenchymal transformation (EMT) has been well characterized in epithelial-to-mesenchymal transition, and transforming growth factor-beta (TGF-β) can initiate and maintain EMT in a variety of biological and pathological systems [14], [15]. The cellular activity of TGF-β is of particular interest in epithelial cells, as it inhibits the G1/S transition of the cell cycle in these cells. However, the same growth factor is the key signaling molecule for EMT, and the role of TGF-β as a key molecule in the development and progression of EMT is well studied [14]–[17]. Smad2/3 are signaling molecules downstream of cell-surface receptors for TGF-β in epithelial-to-mesenchymal transition [16], [17]. Similar to epithelial cells, TGF-β inhibits the G1/S transition of the cell cycle in CECs [18], [19], however, it is not known how TGF-β develops endothelial to mesenchymal transformation and maintains it in CECs. Endothelial-mesenchymal transformation is observed among corneal endothelial dysfunctions such as Fuchs' endothelial corneal dystrophy, pseudoexfoliation syndrome, corneal endotheliitis, surgically-induced corneal endothelial damage, and corneal trauma and it induces the fibroblastic transformation of CECs [20]–[23], suggesting that CECs have the biological potential to acquire endothelial to mesenchymal transformation. The apparent presence of fibroblastic phenotypes in primate CECs and HCECs in culture led us to search for the cause of such phenotypic changes of the cultivated cells and for a means in which to prevent such undesirable cellular changes toward endothelial-mesenchymal transformation.

In the present study, we established primate CEC and HCEC cultures which respectively showed two distinctive phenotypes: 1) normal and 2) fibroblastic. We further characterized the two phenotypes and showed evidence that the use of an inhibitor to TGF-β receptor or BMP-7 abolished the fibroblastic phenotypes of cultivated CECs. Thus, intervention by inhibiting the endothelial to mesenchymal transformation process that occurs during the cultivation of CECs will certainly enable the *in vitro* expansion of cultivated HCECs with a normal phenotype which would be ideal for therapeutic clinical application.

## Materials and Methods

### Ethics Statement

The monkey tissue used in this study was handled in accordance with the ARVO Statement for the Use of Animals in Ophthalmic and Vision Research. The isolation of the tissue was approved by an institutional animal care and use committee of the Nissei Bilis Co., Ltd. (Otsu, Japan) and the Eve Bioscience, Co., Ltd. (Hashimoto, Japan). The human tissue used in this study was handled in accordance with the tenets set forth in the Declaration of Helsinki. A written consent was acquired from the next of kin of all deceased donors regarding eye donation for research. All tissue is recovered under the tenants of the Uniform Anatomical Gift Act (UAGA) of the particular state where the donor was consented and recovered.

### Monkey cornea tissues and Research-grade human cornea tissues

Eight corneas from 4 cynomolgus monkeys (3 to 5 years-of-age; estimated equivalent human age: 5 to 20 years) housed at Nissei Bilis and the Keari Co., Ltd., Osaka, Japan, respectively, were used for the MCECs culture. The cynomolgus monkeys were housed in individual stainless steel cages at Nissei Bilis and Eve Bioscience. Each cage was provided with reverse-osmosis water delivered by an automatic water supply system and supplied with experimental animal diet (PS-A; Oriental Yeast Co., Ltd., Tokyo, Japan). Room temperature was controlled by heating units inside the rooms and was maintained at 18.0–26.0°C. The humidity was maintained at 29.5 to 80.4%. Animals were maintained on a 12∶12-h light:dark cycle (lights on, 7 a.m. to 19 p.m.). For other research purposes, the animals were given an overdose of intravenous pentobarbital sodium for euthanatization. The corneas of cynomolgus monkeys were harvested after confirmation of cardiopulmonary arrest by veterinarians, and then provided for our research. Twenty human donor corneas were obtained from the SightLife^TM^ (Seattle, WA) eye bank, and all corneas were stored at 4°C in storage medium (Optisol; Chiron Vision Corporation, Irvine, CA) for less than 14 days prior to the primary culture.

### Cell culture of monkey CECs (MCECs)

The MCECs were cultivated in modified protocol as described previously [7], [24]. Briefly, the Descemet's membrane including CECs was stripped and digested at 37°C for 2 h with 1 mg/mL collagenase A (Roche Applied Science, Penzberg, Germany). After a digestion at 37°C, the MCECs obtained from individual corneas were resuspended in culture medium and plated in 1 well of a 6-well plate coated with FNC Coating Mix® (Athena Environmental Sciences, Inc., Baltimore, MD). All primary cell cultures and serial passages of the MCECs were performed in growth medium composed of Dulbecco's modified Eagle's medium (Invitrogen Corporation, Carlsbad, CA) supplemented with 10% fetal bovine serum (FBS), 50 U/mL penicillin, 50 μg/mL streptomycin, and 2 ng/mL FGF-2 (Invitrogen). The MCECs were then cultured in a humidified atmosphere at 37°C in 5% CO_2_, and the culture medium was changed every 2 days. When the MCECs reached confluency in 10 to 14 days, they were rinsed in Ca^2+^ and Mg^2+^-free Dulbecco's phosphate-buffered saline (PBS), trypsinized with 0.05% Trypsin-EDTA (Invitrogen) for 5 min at 37°C, and passaged at ratios of 1∶2–4. Cultivated MCECs at passages 2 through 5 were used for all experiments. SB431542 (Merck Millipore, Billerica, MA), a selective inhibitor of transforming growth factor-β (TGF-beta), was tested for the anti-fibroblastic effect.

### Cell culture of HCECs

The HCECs were cultivated in a modified version of the protocol used for the MCECs. Briefly, the Descemet's membrane including CECs was stripped and digested at 37°C for 2 h with 1 mg/mL collagenase A (Roche Applied Science). After a digestion at 37°C, the HCECs obtained from individual corneas were resuspended in culture medium and plated in 1 well of a 12-well plate coated with FNC Coating Mix®. The culture medium was prepared according to published protocols [25], but with some modifications. Briefly, basal culture medium containing OptiMEM-I (Invitrogen), 8% FBS, 5 ng/mL epidermal growth factor (EGF) (Sigma-Aldrich Co., St. Louis, MO), 20 μg/mL ascorbic acid (Sigma-Aldrich), 200 mg/L calcium chloride (Sigma-Aldrich), 0.08% chondroitin sulfate (Wako Pure Chemical Industries, Ltd., Osaka, Japan), and 50 μg/mL of gentamicin was prepared, and the conditioned medium was then recovered after cultivation of inactivated 3T3 fibroblasts. Inactivation of the 3T3 fibroblasts was performed as described previously [26], [27]. Briefly, confluent 3T3 fibroblasts were incubated with 4 μg/mL mitomycin C (MMC) (Kyowa Hakkko Kirin Co., Ltd., Tokyo, Japan) for 2 h at 37°C under 5% CO_2_, and then trypsinized and plated onto plastic dishes at the density of 2×10^4^ cells/cm^2^. The HCECs were cultured in a humidified atmosphere at 37°C in 5% CO_2_, and the culture medium was changed every 2 days. When the HCECs reached confluency in 14 to 28 days, they were rinsed in Ca^2+^ and Mg^2+^-free PBS, trypsinized with 0.05% Trypsin-EDTA for 5 min at 37°C, and passaged at ratios of 1∶2. Cultivated HCECs at passages 2 through 5 were used for all experiments. To test the anti-fibroblastic effect, the cultured HCECs were passaged at the ratio of 1∶2 with medium supplemented with or without SB431542 (0.1, 1, and 10 μM) (Merck Millipore), a neutralizing antibody to TGF-β (500 ng/ml) (R&D Systems, Inc., Minneapolis, MN), Smad3 inhibitor (3 mM) (Merck Millipore), and bone morphogenetic protein (BMP) BMP-7 (10, 100, and 1000 ng/ml) (R&D Systems), and were then evaluated after 1 week.

### Histological examination

For histological examination, cultured MCECs or HCECs on Lab-Tek™ Chamber Slides™ (NUNC A/S, Roskilde, Denmark) were fixed in 4% formaldehyde for 10 min at room temperature (RT) and incubated for 30 min with 1% bovine serum albumin (BSA). To investigate the phenotype of the CECs, immunohistochemical analyses of ZO-1 (Zymed Laboratories, Inc., South San Francisco, CA), a tight junction associated protein, Na^+^/K^+^-ATPase (Upstate Biotechnology, Inc., Lake Placid, NY), the protein associated with pump function, fibronectin (BD, Franklin Lakes, NJ), and actin were performed. ZO-1 and Na^+^/K^+^-ATPase were used as function related markers of the CECs, fibronectin and collagen type 1 were used to evaluate the fibroblastic change, and actin staining was used to evaluate the cellular morphology. The ZO-1, Na^+^/K^+^-ATPase, collagen type 1, and fibronectin staining were performed with a 1∶200 dilution of ZO-1 polyclonal antibody, Na^+^/K^+^-ATPase monoclonal antibody, and fibronectin monoclonal antibody, respectively. For the secondary antibody, a 1∶2000 dilution of Alexa Fluor® 488-conjugated or Alexa Fluor® 594-conjugated goat anti-mouse IgG (Invitrogen) was used. Actin staining was performed with a 1∶400 dilution of Alexa Fluor® 488-conjugated phalloidin (Invitrogen). Cell nuclei were then stained with DAPI (Vector Laboratories, Inc., Burlingame, CA) or propidium iodide (PI) (Sigma-Aldrich). The slides were then inspected by fluorescence microscopy (TCS SP2 AOBS; Leica Microsystems, Wetzlar, Germany). The percentages of Na^+^/K^+^-ATPase- and ZO-1-positive cells that expressed Na^+^/K^+^-ATPase and ZO-1 at the plasma membrane in the *in vivo* condition were counted by a blinded examiner.

### Immunoblotting

For immunoblotting, the cells were washed with PBS and then lysed with radio immunoprecipitation assay (RIPA) buffer (Bio-Rad Laboratories, Hercules, CA) containing Phosphatase Inhibitor Cocktail 2 (Sigma-Aldrich) and Protease Inhibitor Cocktail (Nacalai Tesque, Kyoto, Japan). The lysates were then centrifuged at 15,000 rpm for 10 min at 4°C. The resultant supernatant was collected and the protein concentration of the sample was assessed with the BCA™ Protein Assay Kit (Takara Bio Inc., Otsu, Japan). The proteins were then separated by sodium dodecyl sulfate polyacrylamide gel electrophoresis (SDS-PAGE) and transferred to polyvinylidene fluoride (PVDF) membranes. The membranes were then blocked with 3% non-fat dry milk (Cell Signaling Technology, Inc., Danvers, MA) in TBS-T buffer. The incubations were then performed with the following primary antibodies: Na^+^/K^+^-ATPase (Merck Millipore), ZO-1, GAPDH (Abcam, Cambridge, UK), fibronectin, and Smad2 (Cell Signaling Technology), phosphorylated Smad2 (Cell Signaling Technology), ERK1/2 (BD), phosphorylated ERK1/2 (BD), p38MAPK (BD), phosphorylated p38MAPK (BD) JNK (BD) or phosphorylated JNK (BD) (1∶1000 dilution), and HRP-conjugated anti-rabbit or anti-rabbit IgG secondary antibody (Cell Signaling Technology) (1∶5000 dilution). Membranes were exposed by ECL Advance Western Blotting Detection Kit (GE Healthcare, Piscataway, NJ), and then examined by use of the LAS4000S (Fujifilm, Tokyo, Japan) imaging system.

### Semiquantitative reverse transcriptase polymerase chain reaction (RT-PCR) and quantitative PCR

Total RNA was extracted from CECs and cDNA was synthesized by use of ReverTra Ace® (Toyobo, Osaka, Japan), a highly efficient RT. The same amount of cDNA was amplified by PCR (GeneAmp 9700; Applied Biosystems) and the following primer pairs: GAPDH mRNA, forward (5′-GAGTCAACGGATTTGGTCGT-3′), and reverse (5′-TTGATTTTGGAGGGATCTCG-3′); Na^+^/K^+^-ATPase mRNA, forward (5′-CTTCCTCCGCATTTATGCTCATTTTCTCACCC-3′), and reverse (5′-GGATGATCATAAACTTAGCCTTGATGAACTC-3′); ZO-1 mRNA, forward (5′-GGACGAGGCATCATCCCTAA-3′), and reverse (5′-CCAGCTTCTCGAAGAACCAC-3′); collagen1 mRNA, forward (5′-TCGGCGAGAGCATGACCGATGGAT-3′), and reverse (5′-GACGCTGTAGGT GAAGCGGCTGTT-3′); collagen4 mRNA, forward (5′-AGCAAGGTGTTACAGGATTGGT -3′), and reverse (5′- AGAAGGACACTGTGGGTCATCT -3′); collagen8 mRNA, forward (5′-ATGTGATGGCTGTGCTGCTGCTGCCT -3′), and reverse (5′-CTCTTGGGCCAGGCTCTCCA-3′); fibronectin mRNA, forward (5′-AGATGAGTGGGAACGAATGTCT -3′), and reverse (5′-GAGGGTCACACTTGAATTCTCC -3′); integrin α5 mRNA, forward (5′-TCCTCAGCAAGAATCTCAACAA -3′), and reverse (5′-GTTGAGTCCCGTAACTCTGGTC -3′); integrin β1 mRNA, forward (5′-GCTGAAGACTATCCCATTGACC -3′), and reverse (5′-ATTTCCAGATATGCGCTGTTTT -3′). PCR products were analyzed by agarose gel electrophoresis. Quantitative PCR was performed using the following TaqMan® (Invitrogen) primers: collegen1, Hs00164004_m1; fibronectin, Hs01549976_m1; GAPDH, Hs00266705_g1. The PCR was performed using the StepOne™ (Applied Biosystems) real-time PCR system. GAPDH was used as an internal standard.

### Enzyme-linked immunosorbent assay (ELISA)

Collagen type I of culture medium supernatant of HCECs were measured using ELISA kits for Collagen Type I Alpha 2 (COL1a2) (Uscn Life Science Inc., Wuhan, China) according to the manufacturer's instructions. Culture medium supernatant from HCECs cultured with or without SB431542 were used for each group (n = 5).

### Statistical analysis

The statistical significance (*P*-value) in mean values of the two-sample comparison was determined by use of the Student's t-test. The statistical significance in the comparison of multiple sample sets was analyzed by use of the Dunnett's multiple-comparisons test. Values shown on the graphs represent the mean ± SE.

## Results

### Two distinct phenotypes of primate CECs during cell culture

**Figure 1:**
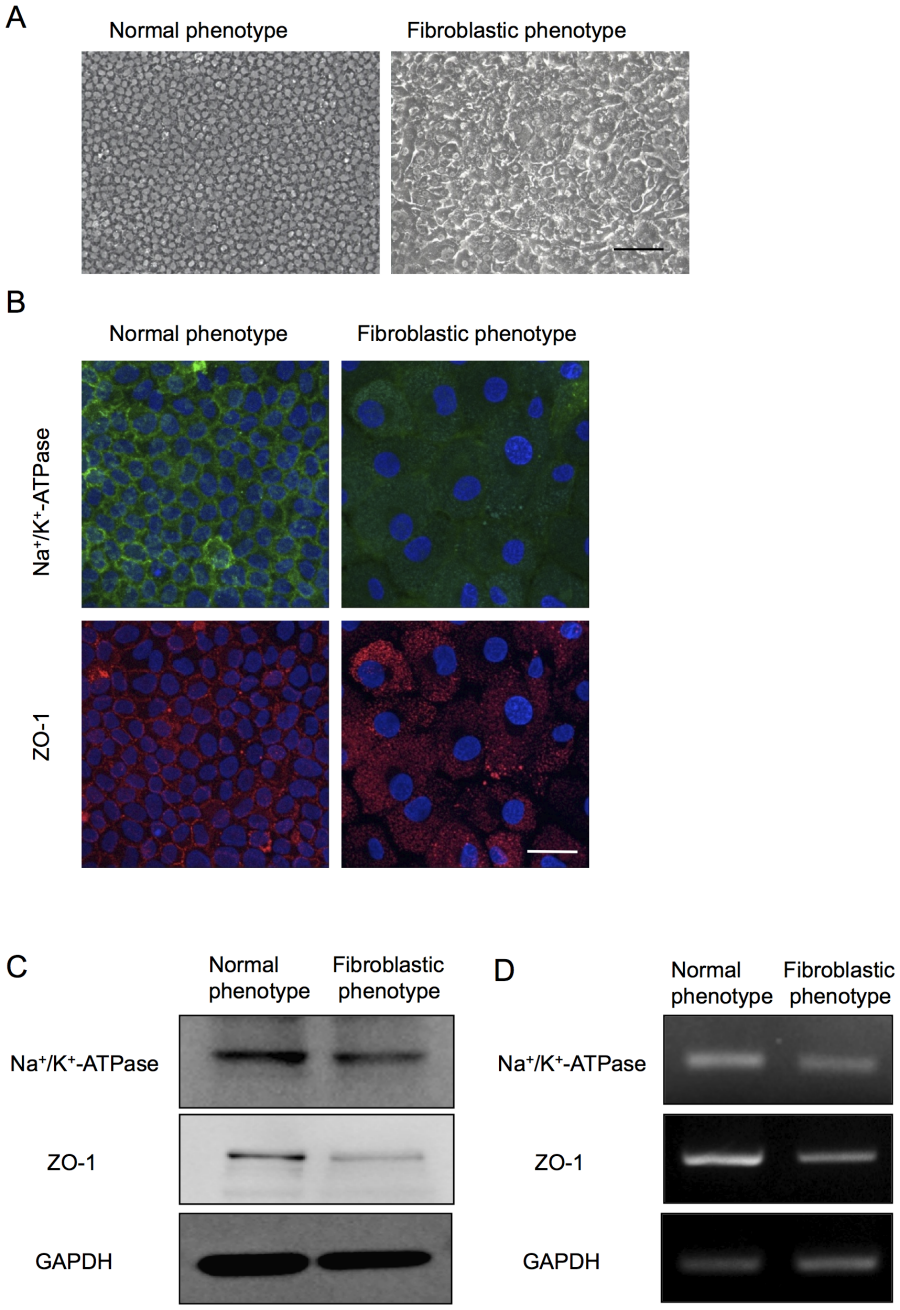
Primate corneal endothelial cells exhibit fibroblastic phenotype and lose functions during cell culture. (A) Cultivated primate CECs demonstrated two distinctive phenotypes; the cells maintained the characteristic polygonal cell morphology and contact-inhibited phenotype (normal phenotype) and the cells showed a fibroblastic cell shape with multi-layering (fibroblastic phenotype). Both phenotypes of the cultured CECs were primary cultured cells. Scale bar: 50 μm. The experiment was performed in triplicate. (B) Na^+^/K^+^-ATPase and ZO-1 at the plasma membrane was preserved in the normal phenotype, while fibroblastic phenotype completely lost the characteristic staining profile of Na^+^/K^+^-ATPase and ZO-1 at the plasma membrane. Scale bar: 100 μm. (C+D) Expression of the Na^+^/K^+^-ATPase and ZO-1 was higher in normal phenotypes than in the fibroblastic phenotypes at both the protein and mRNA levels. Samples were prepared in duplicate. Immunoblotting and semiquantitative PCR were performed in duplicate.

**Figure 2:**
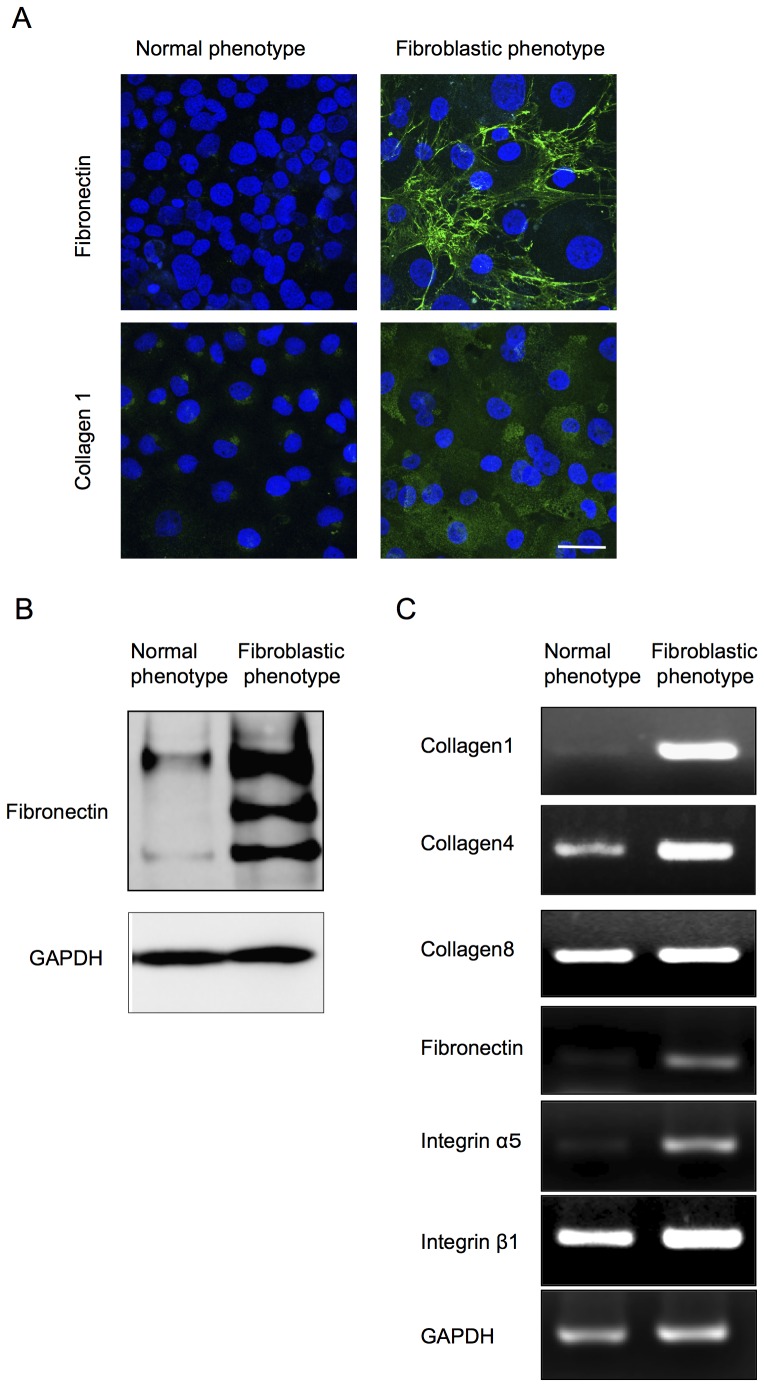
Fibroblastic primate CECs produced an abnormal extra cellular matrix. (A) The fibroblastic phenotype demonstrated excessive ECMs such as fibronectin and collagen type 1, while the normal phenotype completely lost the staining potential. Scale bar: 100 μm. (B) The protein expression level of fibronectin was more strongly upregulated in the fibroblastic phenotype than in the normal phenotype. (C) Semiquantitative PCR analysis showed that the type I collagen transcript [α1(I) mRNA] was abundantly expressed in the fibroblastic phenotypes, while the expression of α1(I) mRNA was reduced in the normal phenotypes. The basement membrane collagen phenotype α1(IV) mRNA was expressed both in normal and fibroblastic phenotypes, yet to a lesser degree in the normal phenotype. Collagen phenotype α1(VIII) mRNA was expressed in both phenotypes at similar levels. Fibronectin and integrin α5 mRNA was observed in the fibroblastic phenotypes, as opposed to the normal phenotypes in which the two transcripts were not expressed. β1 integrin mRNA was expressed in both phenotypes at similar levels. Samples were prepared in duplicate. Immunoblotting and semiquantitative PCR were performed in duplicate.

Of great interest, the primate CECs in culture demonstrated two distinctive phenotypes when determined by cell morphology and the characteristic contact-inhibited phenotype. Although to culture primate CECs in a normal phenotype while maintaining the monolayer contact-inhibited morphology is possible, they often showed morphological fibroblastic change after primary culture following isolation from the cornea, or long-term culture or subculture, if they were once primary cultured in normal morphology (Fig. 1A). The two phenotypes were then tested for the endothelial characteristics; the staining pattern of Na^+^/K^+^-ATPase and ZO-1 at the plasma membrane was well preserved in the normal phenotypes, yet the fibroblastic phenotypes completely lost the characteristic staining profile of Na^+^/K^+^-ATPase and ZO-1 at the plasma membrane (Fig. 1B). Expression of the two functional proteins was found to be much greater in the normal phenotypes than in the fibroblastic phenotypes at both the protein (Fig. 1C) and mRNA levels (Fig. 1D). Comparison of the expression of authentic fibrillar extracellular matrix (ECM) proteins showed that fibroblastic phenotypes demonstrated a fibrillar ECM staining pattern of fibronectin, while the normal phenotypes completely lost the staining potential of fibronectin (Fig. 2A). The protein expression level of fibronectin was more strongly upregulated in the fibroblastic phenotypes than in the normal phenotypes (Fig. 2B). Type I collagen produced by fibroblastic phenotypes demonstrated dual locations, at the ECM and at the cytoplasm. Of interest, the cytoplasmic location of type I collagen appeared to be at the Golgi complex, the intracellular localization of which is essential for secretion, and these findings are similar to the published data [28]. On the other hand, type I collagen staining in the normal phenotypes was not clearly observed (Fig. 2A). RT-PCR analysis was used to determine the expression of major ECM proteins. The type I collagen transcript [α1(I) mRNA] was found to be abundantly expressed in the fibroblastic phenotypes, while the expression of α1(I) mRNA was negligible in the normal phenotypes (Fig. 2C). Unlike the type I collagen transcript, the basement membrane collagen phenotype α1(IV) mRNA was expressed in both the normal and fibroblastic phenotypes, yet to a lesser degree in the normal phenotype. Collagen phenotype α1(VIII) mRNA was expressed in both phenotypes at similar levels. Expression of fibronectin and integrin α5 was observed in the fibroblastic phenotypes, as opposed to the normal phenotypes in which the two transcripts were not expressed (Fig. 2C). On the other hand, β1 integrin mRNA was expressed in both phenotypes at similar levels (Fig. 2C).

**Figure 3:**
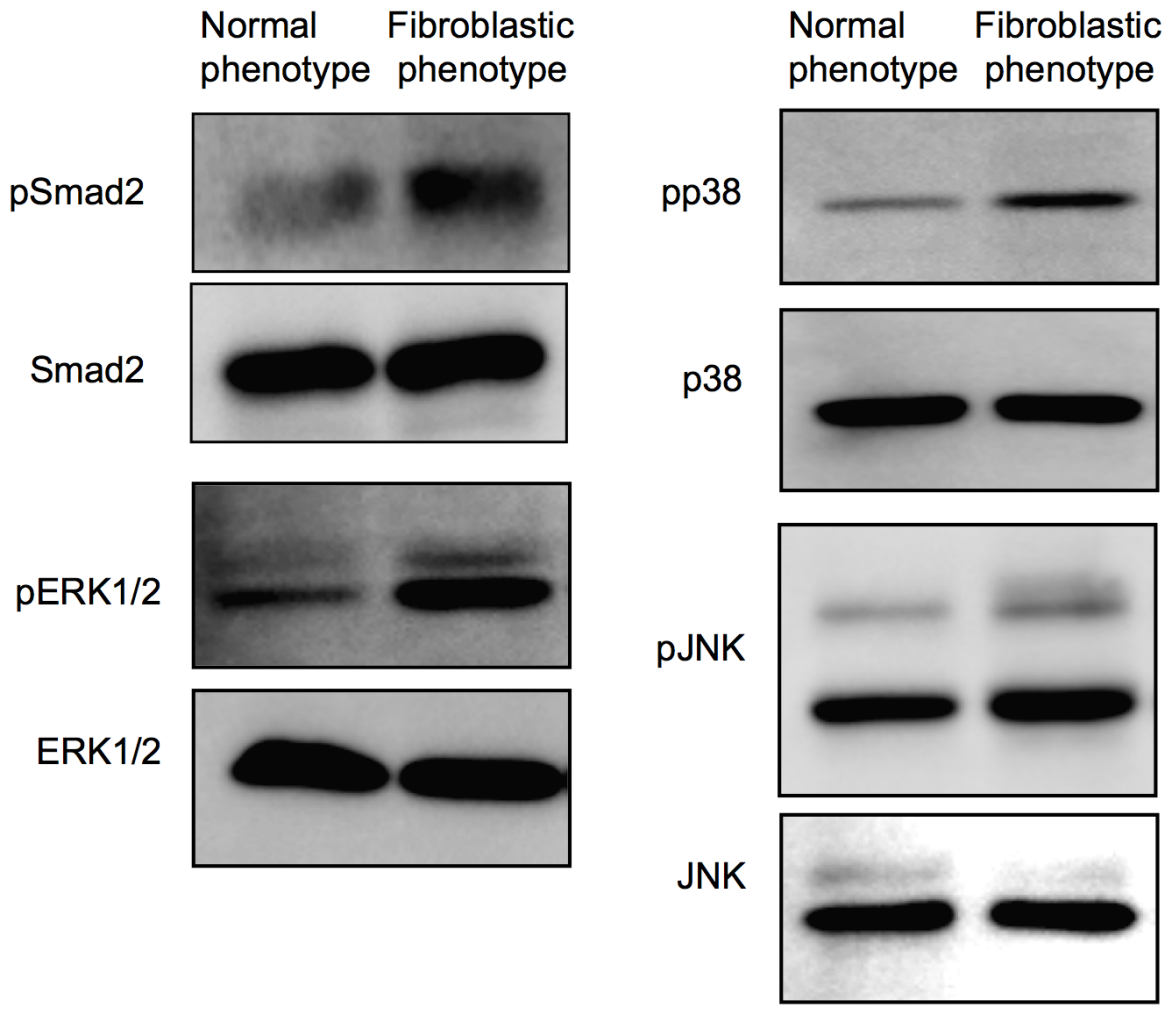
Different activation pattern of fibroblastic change associated pathways in the fibroblastic phenotype of primate CECs. Phosphorylation of Smad2, p38MAPK, and ERK1/2 was promoted in the fibroblastic phenotype compared to that in the normal phenotype, while phosphorylation of JNK was negligible. Samples were prepared in duplicate, and immunoblotting was performed in duplicate.

Next, signaling pathways were determined to elucidate what might cause fibroblastic phenotypes of CECs. Since Smad2, p38, ERK1/2, and JNK are reportedly all involved in the EMT pathway [18]–[20], [29], [30], we therefore tested whether Smad2 and the MAPKs were involved in an endothelial-mesenchymal transformation similar to the EMT observed in epithelial cells (Fig. 3). Phosphorylation of Smad2 was found to be greatly promoted in the fibroblastic phenotypes when compared to that in the normal phenotypes. Phosphorylation of p38 and ERK1/2 was greatly enhanced in the fibroblastic phenotypes, while activation of JNK was negligible. These findings suggested that TGF-β signaling may exert the key role for the fibroblastic transformation of CECs.

### TGF-β-mediated endothelial-mesenchymal transformation and use of TGF-β receptor inhibitor to block it in primate CECs

**Figure 4:**
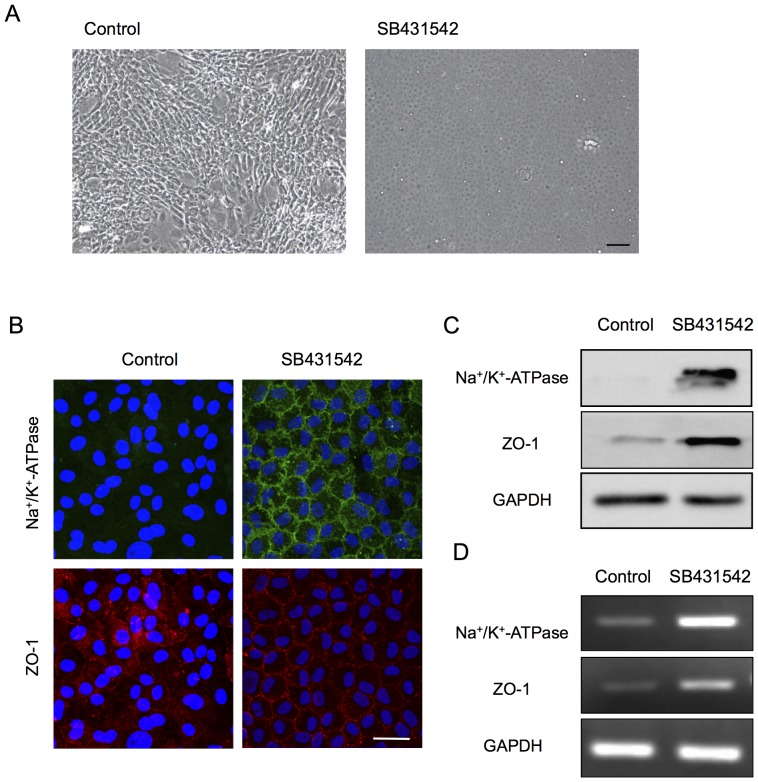
Inhibition of the TGF-β pathway suppressed fibroblastic change and maintained functions. (A) Primate CECs cultured with SB431542 exhibited the authentic polygonal cell shape and contact-inhibited monolayer, while the control CECs exhibited the fibroblastic morphology. Scale bar: 50 μm. (B) SB431542-treated CECs showed the characteristic plasma membrane staining of Na^+^/K^+^-ATPase and ZO-1, while the control CECs lost their staining. Scale bar: 100 μm. (C+D) Expression of Na^+^/K^+^-ATPase and ZO-1 was greatly upregulated in the SB431542-treated fibroblastic phenotypes at both the protein and mRNA levels. Samples were prepared in duplicate. Immunoblotting and semiquantitative PCR were performed in duplicate.

**Figure 5:**
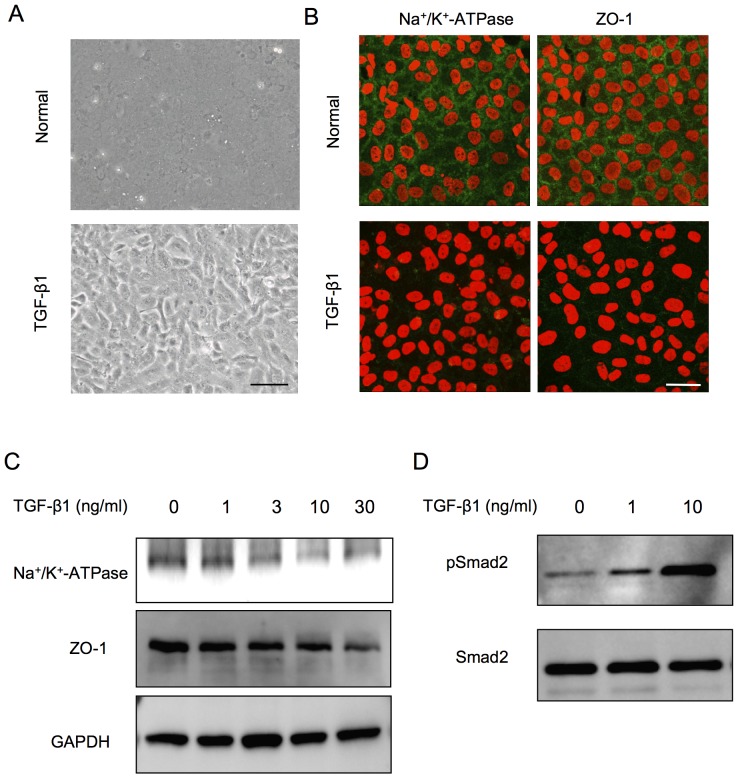
TGFβ induced fibroblastic change and function loss through the activation of the Smad signaling pathways. (A) Normal phenotype primate CECs were transformed to fibroblastic cells when exposed to the exogenous TGF-β1 (10 ng/ml). Scale bar: 50 μm. (B) The staining pattern of Na^+^/K^+^-ATPase and ZO-1 at the plasma membrane of the normal phenotypes was greatly reduced upon exposure to TGF-β1 (10 ng/ml). Scale bar: 100 μm. (C) TGF-β1 reduced the expression of Na^+^/K^+^-ATPase and ZO-1 at protein levels dose-dependently. (D) Phosphorylation of Smad2 was increased in a concentration-dependent manner. Samples were prepared in duplicate, and immunoblotting was performed in duplicate.

The findings shown in Fig. 3 led us to directly test whether SB431542, the TGF-β receptor inhibitor, was able to block the EMT process observed in the fibroblastic phenotypes. Phase contrast imaging demonstrated that primate CECs cultured in the presence of SB431542 exhibited the authentic polygonal cell shape and contact-inhibited monolayer, while the control CECs exhibited the fibroblastic morphology (Fig. 4A). Moreover, the SB431542-treated CECs showed the characteristic plasma membrane staining of Na^+^/K^+^-ATPase and ZO-1, while the control CECs lost their staining, suggesting that endothelial functions were maintained in the SB431542-treated cells (Fig. 4B). Furthermore, the expression of Na^+^/K^+^-ATPase and ZO-1 was strongly upregulated in the SB431542-treated fibroblastic phenotypes at both the protein (Fig. 4C) and mRNA levels (Fig. 4D). These data further confirmed that TGF-β might be the direct mediator of the endothelial to mesenchymal transformation observed in primate CEC cultures. Therefore, we tested whether the normal phenotypes were transformed to fibroblastic cells when exposed to the exogenous TGF-β, as in the findings shown in Fig. 5A. Of interest, the staining pattern of Na^+^/K^+^-ATPase and ZO-1 at the plasma membrane of the normal phenotypes was greatly reduced upon exposure of polygonal cells to TGF-β (Fig. 5B). The growth factor also markedly reduced the expression of the two proteins at protein levels in a concentration-dependent manner (Fig. 5C), while phosphorylation of Smad2 was greatly increased in a concentration-dependent manner (Fig. 5D). These data suggest that even the normal phenotypes of primate CECs are prone to acquire fibroblastic phenotypes in response to TGF-β-stimulation.

### Two distinct phenotypes of HCEC cultures and the use of TGF-β receptor inhibitor to block endothelial-mesenchymal transformation

**Figure 6:**
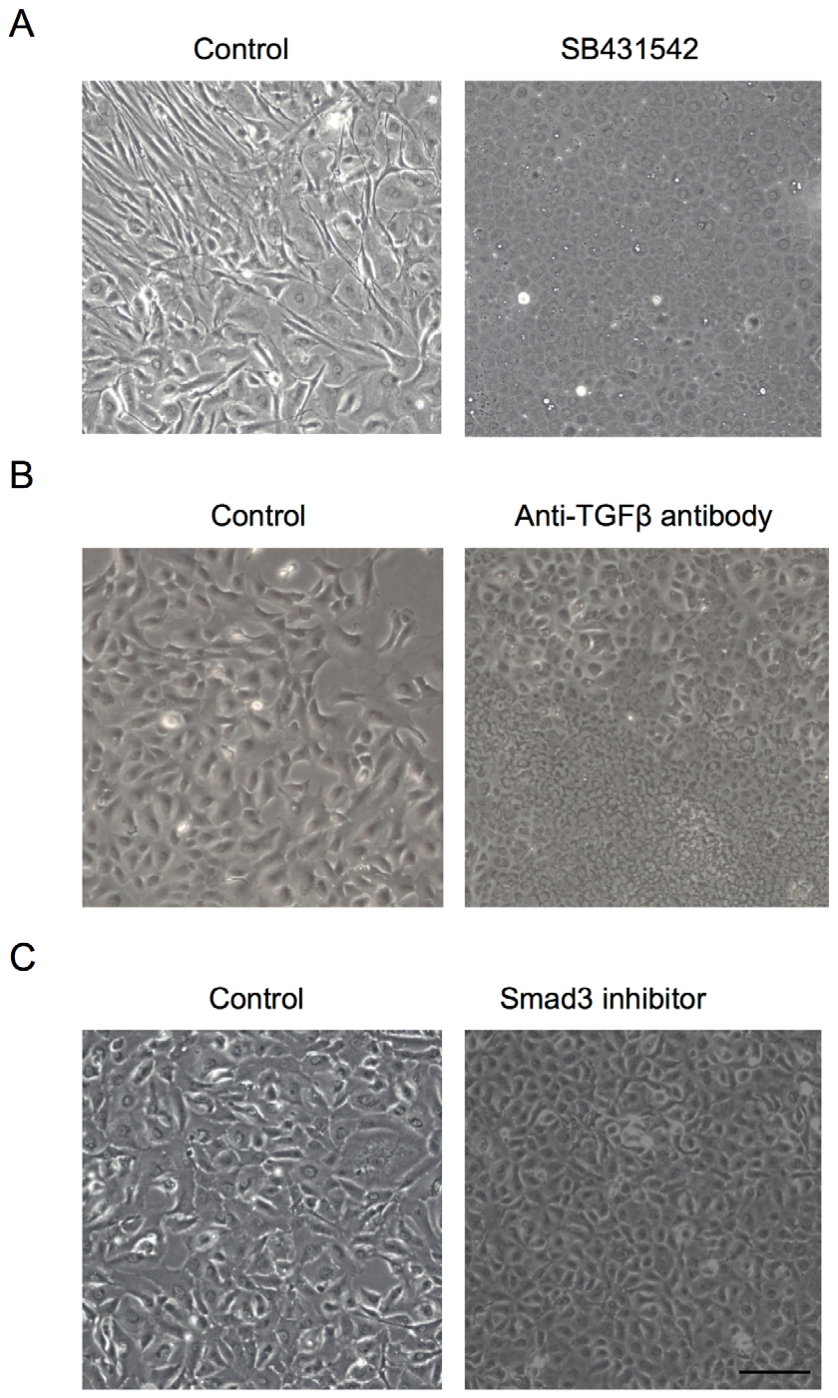
Inhibition of the TGFβ pathway suppressed the fibroblastic change of HCECs. (A) HCECs cultured with SB431542 (1 μM) exhibited the hexagonal cell shape and contact-inhibited monolayer, while the control CECs exhibited the fibroblastic morphology. (B+C) Both neutralizing antibody to TGF-β (500 ng/ml) and Smad3 inhibitor (3 mM) blocked cells from acquiring fibroblastic phenotypes. Scale bar: 50 μm. The experiment was performed in duplicate.

**Figure 7:**
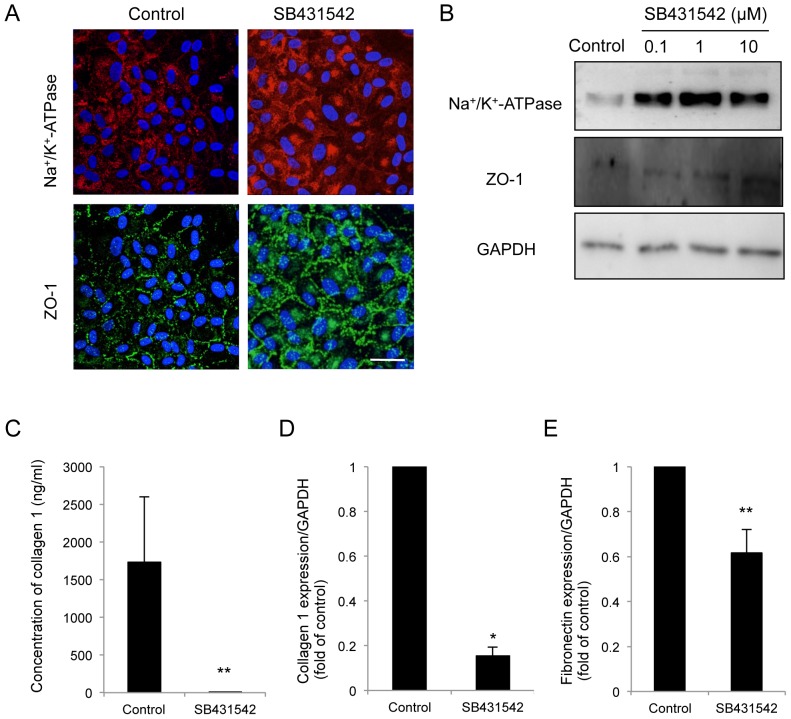
SB431542 maintained the functions and suppressed the fibroblastic change of HCECs. (A+B) Blocking the TGF-receptor signaling by SB431542 (A: 1 μM, B: 0.1, 1, and 10 μM) enabled the subcellular localization of Na^+^/K^+^-ATPase and ZO-1 at the plasma membrane and their protein expression to be maintained. Scale bar: 100 μm. (C) ELISA assay revealed that SB431542 significantly downregulated the secretion of type I collagen to the culture supernatant. **P<0.05. (D+E) Quantitative PCR showed that SB431542 significantly reduced the expression of type I collagen and fibronectin at the mRNA level. *p<0.01, ** *p*<0.05. Samples were prepared in duplicate. Immunoblotting, ELISA, and quantitative PCR were performed in duplicate.

The interesting findings observed in primate CECs led us to further study whether HCECs were subjected to the similar undesirable prerequisite cellular changes leading to endothelial-mesenchymal transformation. Of great interest, cultivated HCECs lost the characteristic contact-inhibited monolayer and polygonal phenotypes, and acquired fibroblastic cell morphology like primate CECs (Fig. 6A). However, consistent with the primate CECs when the CECs were cultivated with the specific inhibitor to the TGF-β receptor (SB431542), the inhibitor was able to block alteration of the cell shape to fibroblastic phenotypes. Similar to the inhibitory effect of SB431542 on fibroblastic phenotypes, both neutralizing antibody to TGF-β (Fig. 6B) and Smad3 inhibitor (Fig. 6C) also blocked cells from acquiring fibroblastic phenotypes. We then tested whether SB431542 was able to maintain endothelial function. The findings shown in Fig. 7A and Fig. 7B demonstrated that blocking the TGF-β receptor signaling enabled the subcellular localization of Na^+^/K^+^-ATPase and ZO-1 at the plasma membrane and their protein expression to be maintained. Of great importance, ELISA assay revealed that SB431542 markedly downregulated the secretion of type I collagen to the culture supernatant (Fig. 7C). Coincidentally, SB431542 markedly reduced the expression of type I collagen and fibronectin at the mRNA level (Fig. 7D, E).

### Use of BMP-7 to suppress fibroblastic changes and maintain endothelial functions

**Figure 8:**
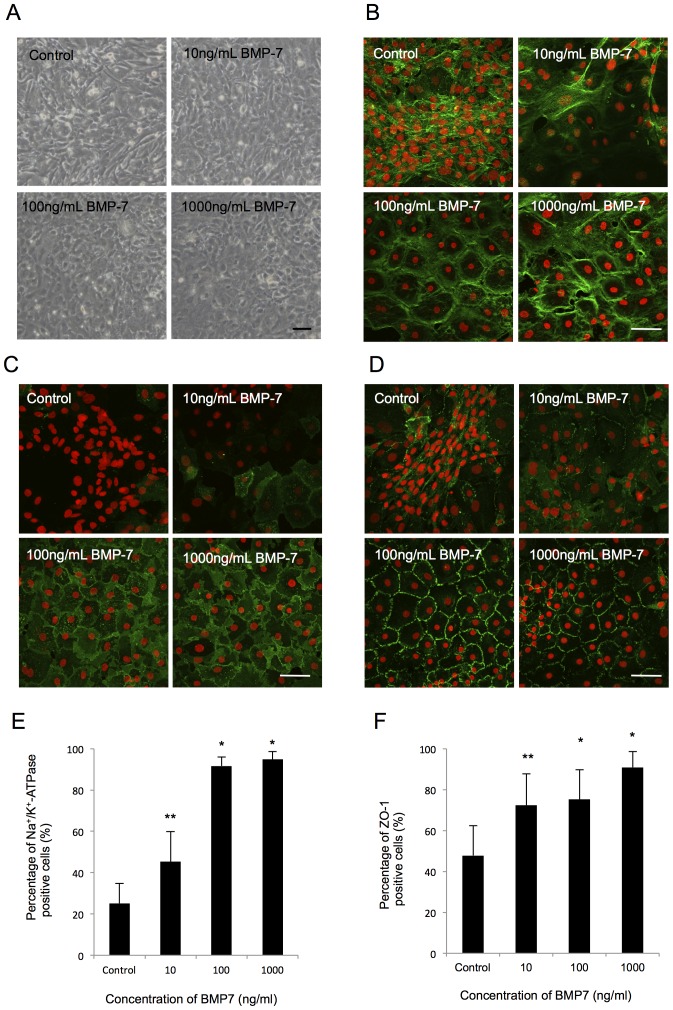
BMP7 suppressed fibroblastic change and maintained the functions of HCECs. (A) The elongated cell shapes of the fibroblastic phenotypes were reversed to a polygonal cell morphology in response to the presence of BMP-7 in a concentration-dependent manner. Scale bar: 50 μm. (B) BMP-7 enabled normal hexagonal cell morphology and actin cytoskeleton distribution at the cortex to be maintained. Scale bar: 100 μm. (C+D) BMP-7 maintained the subcellular localization of Na^+^/K^+^-ATPase and ZO-1at the plasma membrane. Scale bar: 100 μm. (E+F) The percentages of both Na^+^/K^+^-ATPase and ZO-1 positive cells treated with BMP-7 were significantly higher than in the control. * *p*<0.01, ** *p*<0.05. The experiment was performed in duplicate.

Bone morphogenetic protein-7 (BMP-7) promotes MET and specifically inhibits the TGF-β-mediated epithelial-to-mesenchymal transition. Thus, that molecule has been used to antagonize the EMT process [31]–[34]. We therefore tested whether BMP-7 was able to antagonize the prerequisite changes of HCECs. The fibroblastic HCECs were treated with BMP-7 in a concentration ranging from 10 to 1000 ng/ml. Of important note, the elongated cell shapes of the fibroblastic phenotypes were reversed to the polygonal cell morphology in response to the presence of BMP-7 in a concentration-dependent manner (Fig. 8A). BMP-7 enabled the hexagonal cell morphology and actin cytoskeleton distribution at the cortex to be maintained (Fig. 8B), similar to that observed in normal CECs [35], and it also maintained the subcellular localization of Na^+^/K^+^-ATPase (Fig. 8C) and ZO-1 (Fig. 8D) at the plasma membrane. Thus, BMP-7 at the concentration of 1000 ng/ml was able to maintain CECs in polygonal and contact-inhibited phenotypes with a positive expression of function-related markers (Fig. 8E, F).

## Discussion

Corneal endothelial dysfunction accompanied by visual disturbance is a major indication for corneal transplantation surgery [36], [37]. Though corneal transplantation is widely performed for corneal endothelial dysfunction, researchers are currently seeking alternative methods to restore healthy corneal endothelium. The fact that corneal endothelium is cultured and stocked as ‘master cells’ from young donors allows for the transplantation of CECs with high functional ability and for an extended period of time. In addition, an HLA-matching transplantation to reduce the risk of rejection [38], [39] and overcoming the shortage of donor corneas might be possible. Tissue bioengineering is a new approach to develop treatments for patients who have lost visual acuity [40]. To date, there are two methods that utilize bioengineering approaches: 1) use of cultured donor HCECs adhered on bioengineered constructs [4], [5], [7], [9], and 2) transplantation of cultivated HCECs into the anterior chamber [11], [41]–[43]. Regardless of which of the two methods is applied to clinical settings, establishment of an efficient cultivation technique for HCECs is essential and inevitable [44]. Many researchers have noticed that establishing a consistent long-term culture of HCECs is challenging [40]. Although the successful cultivation of HCECs has been reported by several groups, the procedures involved in the isolation and subsequent cultivation protocols varied greatly between laboratories [44]. One of the most difficult problems is that HCECs are vulnerable to undergoing massive fibroblastic change over each passage [40]. Therefore, it is essential to find means to circumvent the spontaneous transformation of the CECs in order to maintain the physiological phenotypes for the subsequent use for transplantation.

Transformation of endothelial cells to fibroblastic cells is designated as endothelial- mesenchymal transformation. Such transformation is triggered by TGF-β via the Smad2/3 pathway [16]. Endothelial-mesenchymal transformation causes the loss of the characteristic endothelial phenotypes, such as loss of the contact-inhibited monolayer and loss of the apical junctional proteins at the plasma membrane. Furthermore, it causes induction of fibrillar proteins such as type I collagen and fibronectin. In this present study, we demonstrated that the fibroblastic phenotypes of cultivated CECs greatly lost the endothelial characteristics; expression of Na^+^/K^+^-ATPase and ZO-1 was markedly reduced and their subcellular localization was in the cytosol rather than the authentic plasma membrane location. Furthermore, fibroblastic phenotypes markedly enhance the production of fibrillar ECM proteins (type I collagen, fibronectin, and integrin α5) rather than basement membrane phenotypes (type IV and VIII collagens). The presence of such undesirable cells will greatly hamper the success of transplantation of cultivated cells in the clinical setting. Therefore, it is crucial to determine what causes the phenotypic changes and how to intervene in such endothelial-mesenchymal transformation processes of the cultivated CECs. The fact that phosphorylation of Smad2/3 was greatly enhanced in the fibroblastic phenotypes led us to conclude that the fibroblastic phenotypes in both primate and HCECs are mediated by TGF-β signaling. Therefore, we employed a specific inhibitor to the TGF-β receptor (SB431542) [45] to block the endothelial-mesenchymal transformation process observed in the fibroblastic phenotypes. SB431542 completely abolished the undesirable cellular changes, and when either primate or HCEC cultures were treated with SB431542, the prerequisite change of cells to fibroblastic phenotypes was completely abolished. Simultaneously, the characteristic subcellular location of ZO-1 and Na^+^/K^+^-ATPase is resumed at the plasma membrane and the expression of the two proteins is greatly increased at both mRNA and protein levels, suggesting that the barrier and pump functions in these cultures is intact. Moreover, we found that the production of fibrillar ECM proteins was greatly reduced. We further tested the effect of BMP-7, a well-known anti-EMT agent [31], [34], to reverse the fibroblastic phenotypes of HCECs. BMP-7 also reversed the fibroblastic phenotypes to the normal endothelial cells with contact-inhibited monolayer and characteristic endothelial adhesion. Taken together, both SB431542 and BMP-7 can be powerful tools to maintain the normal endothelial phenotypes of the cultivated CECs, thus leading to a successful subsequent transplantation.

In conclusion, our findings showed that the use of the inhibitor to TGF-β receptor (SB431542) and/or anti-EMT molecules (BMP-7) enables HCECs to grow with maintaining normal physiological function (i.e., barrier and pump function). Although more extensive future studies would be beneficial, we have not observed any obvious adverse effects of continuous SB431542 or BMP-7 treatment on morphology and functions, even after several numbers of passages. This present study may prove to be the substantial protocol to provide the efficient *in vitro* expansion of HCECs. In addition, this novel strategy of inhibition of fibroblastic change during cultivation may ultimately provide clinicians with a new therapeutic modality in regenerative medicine, not only for the treatment of corneal endothelial dysfunctions, but also for a variety of pathological diseases in general.
